# Massive Bilateral Exudative Retinal Detachment Revealing Pheochromocytoma: A Case Report

**DOI:** 10.7759/cureus.76939

**Published:** 2025-01-05

**Authors:** Hamza Lazaar, Taha Boutaj, Zineb FIlali, Boutayna Azarkan, Mourad Labhar, Saad Benchekroun, Abdellah Amazouzi, Lalla Ouafa Cherkaoui

**Affiliations:** 1 Ophthalmology, Hôpital des Spécialités de Rabat, Rabat, MAR; 2 Faculty of Medicine, Centre Hospitalo-Universitaire Ibn Sina, Rabat, MAR; 3 Ophthalmology, Hôpital des Specialités, Université Mohammed V de Rabat, Rabat, MAR

**Keywords:** choroidopathy, exudative, malignant hypertension, retinal detachment, secondary hypertension

## Abstract

Hypertension is a leading cause of cardiovascular morbidity and premature death that can result in significant end-organ damage, including to the eyes. Malignant hypertension, defined by a blood pressure above 200/120 mmHg, can lead to advanced retinopathy with complications such as exudative retinal detachment. We present a case of a 28-year-old woman who developed bilateral blindness due to malignant hypertension caused by a pheochromocytoma, an uncommon tumor that secretes catecholamines. Initial ophthalmic examination revealed severe hypertensive retinopathy and bilateral exudative retinal detachment; the diagnosis was confirmed by elevated urinary catecholamines and a CT scan showing an adrenal mass. This case underscores the importance of identifying ocular manifestations of hypertension as indicators of life-threatening systemic conditions. Prompt diagnosis and management, including antihypertensive treatment and surgical intervention, are critical for such patients.

## Introduction

Hypertension is the major cause of cardiovascular morbidity and premature deaths worldwide. In 2010, its prevalence was estimated at around 31%, impacting over a billion individuals worldwide [1].

The International Society of Hypertension (ISH) defines malignant hypertension as a marked elevation in blood pressure (commonly > 200/120 mmHg) accompanied by advanced bilateral retinopathy, such as hemorrhages, cotton wool spots, or papilledema [2].

Secondary hypertension is caused by underlying conditions that can often be treated to normalize blood pressure. Common causes include kidney diseases (e.g., chronic kidney disease, renal artery stenosis), endocrine disorders (e.g., hyperaldosteronism, pheochromocytoma, Cushing’s syndrome), and obstructive sleep apnea. It can also result from certain medications (e.g., nonsteroidal anti-inflammatory drugs (NSAIDs), contraceptives) or substances (e.g., alcohol, cocaine). Rare causes include vascular anomalies (e.g., aortic coarctation) and genetic syndromes (e.g., Liddle syndrome). Identifying secondary hypertension is crucial, especially in cases of severe, resistant, or early-onset hypertension [3].

Secondary causes should always be considered in cases of hypertension in young patients [4].

We present the exceptional case of a young patient whose presentation of pheochromocytoma was bilateral blindness secondary to malignant hypertension with massive bilateral exudative retinal detachment.

## Case presentation

We present the case of a 28-year-old female patient with no notable medical or surgical history who was admitted to the ophthalmology emergency department for profound bilateral visual acuity reduction and intense headaches for two days. The ophthalmological examination revealed a visual acuity reduced to motion perception. Intraocular pressure measured with Goldman tonometry was 13 mmHg for both eyes. The pupillary reflex was sluggish and ocular motility was preserved. Biomicroscopic examination showed bilateral conjunctival hemorrhage with chemosis. The cornea was clear with a normal anterior chamber depth, a normal iris structure, and a clear lens in both eyes.

**Figure 1:**
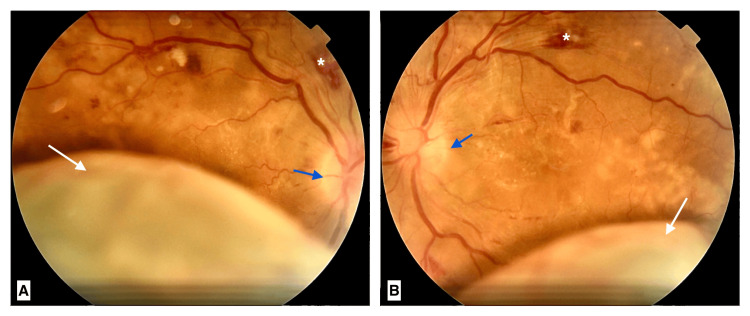
Fundus photography - right eye (A) and left eye (B). Findings included arterial narrowing, flame-shaped hemorrhages (white star), cotton wool spots, perimacular exudates, papilledema (blue arrow), and inferior exudative retinal detachment (white arrow).

Fundus examination revealed bilateral arterial narrowing, flame-shaped hemorrhages along the temporal arcades and peripapillary area, cotton wool spots, and perimacular exudates along with papilledema. The picture was complicated by an inferior exudative retinal detachment, more significant in the right eye (Figure 1). A systemic blood pressure measurement found 240/140 mmHg. The clinical presentation corresponded to bilateral hypertensive retinopathy and choroidopathy complicated by exudative retinal detachment.

**Figure 2:**
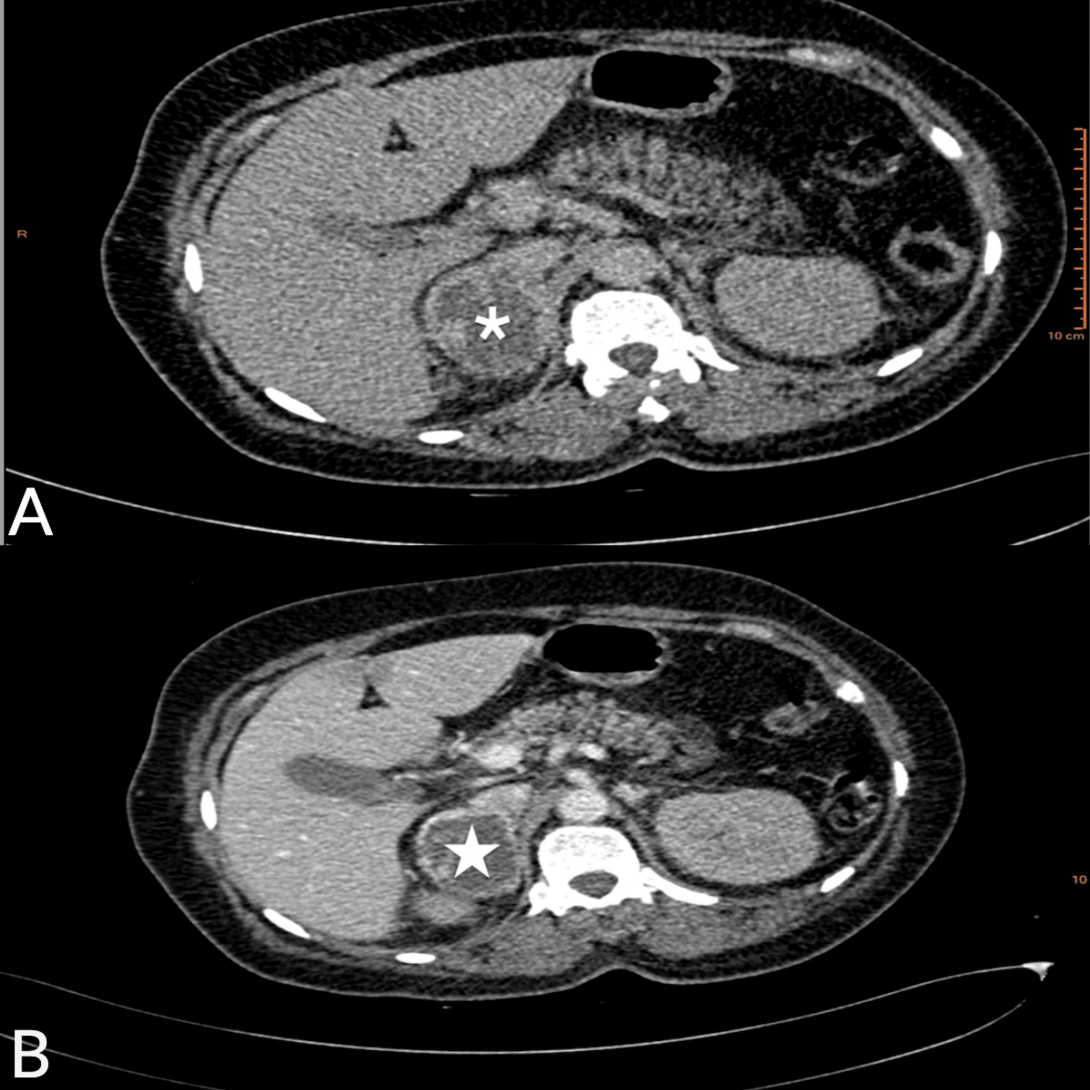
CT scan before (A) and after contrast injection (B) revealed a right adrenal mass (white star) of 4 cm, solid-cystic, with the tissue component enhancing after contrast injection.

Rapid initiation of intravenous antihypertensive treatment was indicated. A workup was requested, including a normal ionogram and renal function; urinary catecholamines showed values of 1245 µg/24h (3.5x normal) for metanephrines and normetanephrine 687 µg/24h (1.5x normal). A thoracoabdominal CT scan before and after contrast injection revealed a right adrenal mass of 4 cm, solid-cystic, with the tissue component enhancing after contrast injection (Figure 2). The diagnosis of pheochromocytoma was therefore suspected.

The patient received antihypertensive treatment consisting of intravenous nicardipine and oral beta-blockers and was transferred to general surgery for tumor resection. However, she was lost to follow-up and did not return for subsequent check-ups.

## Discussion

Malignant hypertension refers to an acute rise in systemic blood pressure associated with advanced bilateral retinopathy. The term was initially introduced by Volhard and Fahr in 1914 [5] and later adopted by Keith et al. in 1928 [6]. Its incidence is rare and variable depending on geographic location, with about one case for 100,000 inhabitants per year in Europe [7].

Malignant hypertension may manifest through three anatomical-clinical entities: hypertensive retinopathy, optic neuropathy, and more rarely choroidopathy. Hypertensive retinopathy may present with peripapillary flame-shaped hemorrhages, blurring of the optic disk margin, pronounced papilledema with signs of venous stasis, and macular exudates [8]. Optic neuropathy depends less on severity and more on the chronicity of hypertension. It presents as optic disc swelling or optic nerve pallor in patients with chronic hypertension [9].

As in our case, hypertensive choroidopathy can cause focal detachment of the pigmented epithelium, leading to exudative retinal detachment [10]. This is due to a marked rise in systemic blood pressure exceeding the capacity for autonomic regulation, leading to obstruction by fibrin and platelet deposits. This obstruction affects the choroidal arteries and choriocapillaries, causing necrosis of the overlying retinal pigment epithelium. Consequently, exudation occurs, leading to an exudative retinal detachment [11].

Similar cases of retinal detachment have been previously documented in association with conditions such as preeclampsia [12], eclampsia [13], hemolysis, elevated liver enzymes, and low platelet count (HELLP) syndrome [14], primary pulmonary hypertension [15], or acute kidney injury [16].

Pheochromocytoma is an uncommon tumor that secretes catecholamines and typically arises from the chromaffin cells of the adrenal medulla. Around 70% of pheochromocytomas are sporadic, while the remaining 30% are linked to hereditary diseases such as multiple endocrine neoplasia type 2, von Hippel-Lindau (VHL) syndrome, or neurofibromatosis type 1 [17].

Arterial hypertension is the primary clinical sign of pheochromocytoma reported in 95% of patients [18]. It can manifest in two clinical forms: sustained hypertension and paroxysmal hypertension. Ocular signs commonly associated with pheochromocytoma are those seen in hypertensive retinopathy such as microaneurysms, retinal hemorrhages, cotton wool spots, and venous bleeding [18]. Additionally, cases of bilateral papillopathy have been reported in the literature as a presenting sign of pheochromocytoma [19]. However, bilateral retinal detachment has never been documented in association with this condition.

Our case is rare due to the severity of the detachment and the presence of all three characteristics - retinopathy, choroidopathy, and neuropathy - as well as the etiology since this is, to our knowledge, the first case of pheochromocytoma revealed by bilateral exudative detachment due to hypertensive choroidopathy. 

Malignant hypertension is considered a hypertensive emergency, mandating immediate and controlled blood pressure reduction to a safe level as well as treatment of the etiology in cases of secondary hypertension, as in our case, where surgery for pheochromocytoma is necessary.

In summary, the report details the case of a young woman with massive bilateral retinal detachment secondary to hypertensive chorioretinopathy due to a pheochromocytoma. 

## Conclusions

This case highlights the critical importance of recognizing ocular manifestations of systemic hypertension, particularly in life-threatening conditions such as pheochromocytoma. The exceptional presentation of malignant hypertension with bilateral massive exudative retinal detachment underscores the need for comprehensive diagnostic workups in young patients presenting with hypertensive ocular findings. Early identification and management of the underlying etiology, such as surgical resection of pheochromocytoma, are vital to prevent further systemic and ocular complications. This report emphasizes the ophthalmologist's pivotal role in diagnosing and initiating timely intervention for severe systemic diseases.
